# Specialist physician knowledge of chronic kidney disease: A comparison of internists and family physicians in West Africa

**DOI:** 10.4102/phcfm.v4i1.319

**Published:** 2012-05-29

**Authors:** Emmanuel I. Agaba, Patricia A. Agaba, Musa Dankyau, Maxwell O. Akanbi, Comfort A. Daniyam, Edith N. Okeke, Antonios H. Tzamaloukas

**Affiliations:** 1 Department of Medicine, Jos University Teaching Hospital, Jos, Nigeria; 2 Department of Family Medicine, Bingham University Teaching Hospital, Jos, Nigeria; 3 Renal Section, New Mexico Veterans Affairs Health Care System, USA; 4 Department of Medicine, University of New Mexico School of Medicine, Albuquerque, USA

## Abstract

**Background:**

Postgraduate training is aimed at equipping the trainee with the necessary skills to practise as an expert. Non-nephrology specialist physicians render the bulk of pre-end-stage renal disease care for patients with chronic kidney disease (CKD). We sought to ascertain the knowledge of CKD amongst non-nephrology specialist physicians who serve as trainers and examiners for a training, accrediting and certifying body in postgraduate medicine in West Africa. We also compared the knowledge of family physicians and non-nephrology internists.

**Methods:**

Self-administered questionnaires were distributed to non-nephrology specialist physicians who serve as examiners for the West African College of Physicians.

**Results:**

Only 19 (27.5%) of the respondents were aware of the Kidney Disease Outcomes Quality Initiatives guidelines for CKD management. Twenty five (36.2%) of the respondents had adequate knowledge of CKD. There was no significant difference in the proportion of family physicians and non-nephrology internists who had adequate knowledge of CKD (27.3% vs. 40.4% respectively; *p* = 0.28). Hypertension and diabetes mellitus were identified by all of the physicians as risk factors for CKD. Non-nephrology internists more frequently identified systemic lupus erythematosus as a risk factor for CKD, urinalysis with microscopy as a laboratory test for CKD evaluation, and bone disease as a complication of CKD than family physicians.

**Conclusion:**

There is a lack of adequate CKD knowledge amongst non-nephrology specialist physicians, since many of them are unaware of the CKD management guidelines. Educational efforts are needed to improve the knowledge of CKD amongst non-nephrology specialist physicians. Guidelines on CKD need to be widely disseminated amongst these physicians.

## Abstrait

**Connaissances des spécialistes des maladies rénales chroniques: Une comparaison des internistes et des médecins de famille en Afrique de l'Ouest**

**Contexte:**

La formation de troisième cycle vise à donner à l’étudiant les compétences nécessaires pour exercer en tant qu'expert. Les spécialistes hors néphrologie réalisent le gros de la prise en charge des maladies rénales en phase terminale pour les patients atteints de maladie rénale chronique (MRC). Nous avons cherché à déterminer les connaissances sur la MRC chez les spécialistes hors néphrologie qui sont formateurs et examinateurs pour une structure de formation, d'accréditation et de certification en médecine de troisième cycle en Afrique de l'Ouest. Nous avons également comparé les connaissances des médecins de famille et des internistes hors néphrologie.

**Méthodes:**

Des questionnaires auto-administrés ont été distribués à des spécialistes hors néphrologies qui occupaient des fonctions d'examinateurs pour le Collège ouest-africain des médecins.

**Résultats:**

Seulement 19 (27.5%) des sondés connaissaient les directives de la Kidney Disease Outcomes Quality Initiative pour la prise en charge de la MRC. Vingt-cinq (36.2%) d'entre eux disposaient de connaissances adéquates de la MRC. Aucune différence significative n'a été constatée dans la proportion de médecins de famille et d'internistes hors néphrologie qui avaient des connaissances adéquates de la MRC (27.3%, contre 40.4% respectivement; *p* = 0.28). L'hypertension et le diabète non insulinodépendant ont été identifiés par tous les médecins comme des facteurs de risque de la MRC. Les internistes hors néphrologie ont identifié plus fréquemment que les médecins de famille le lupus érythémateux disséminé comme un facteur de risque de la MRC, l'analyse d'urine au microscope comme test de laboratoire pour le dépistage de la MRC et la maladie osseuse comme une complication de la MRC.

**Conclusion:**

Les connaissances adéquates de la MRC sont insuffisantes chez les spécialistes hors néphrologie, puisque bon nombre ne connaissaient pas les directives de prise en charge de la MRC. Des efforts d’éducation sont nécessaires afin d'améliorer les connaissances de la MRC chez les spécialistes hors néphrologie. Les directives sur la MRC doivent être largement diffusées auprès de ces médecins.

## Introduction

### Key focus

There is a pandemic of chronic kidney disease (CKD), with the majority of affected individuals being under-diagnosed and under-treated.^1^ It has been estimated that CKD affects approximately 10% of the general population. It is usually characterised by progression to end-stage renal disease, and is associated with increased cardiovascular morbidity and mortality. CKD has been shown to be an independent predictor of mortality, with increasing effect as the stages progress.^2^ However, timely medical intervention which can slow progression of CKD and prevent end-stage renal disease has been advocated, since the cost of end-stage renal disease programmes is prohibitive. The overall cost of CKD treatment per person per year in 2008 in the United States of America (USA) was estimated to have been between $16 738 and $19 752.^3^ The quality of life of patients on dialysis is poor, and the annual cost of haemodialysis in the USA exceeds $60 000 per patient.^4^

In the developing world renal replacement therapy is constrained by cost and lack of technological advancements. We previously reviewed the practice and cost of haemodialysis in a teaching hospital in Nigeria,^5^ finding that most patients presenting in end-stage renal disease at this hospital – as in many others in Nigeria – are not dialysed because they cannot afford it. Furthermore, dialysis sessions last as long as nine hours due to repeated breakdown of antiquated dialysis machines. The National Kidney Foundation's Kidney Disease Outcomes Quality Initiatives clinical practice guidelines emphasise the need for early detection and management of CKD in order to prevent end-stage renal disease and its consequences.^6^

### Background

Most individuals with CKD present late to nephrologists – in some instances only when in uraemia.^7^ We previously reported on the grim picture in Nigeria, where all patients needing dialysis in a teaching hospital were presenting to the nephrologist for the first time in overt uraemia.^8^ This is largely due to late referrals and the dearth of nephrologists worldwide. Taking the USA as an example of the developed world, there were 5500 full-time practising nephrologists as at 2009.^9^ The situation is even worse in the developing countries: as at 2009, Nigeria (population over 140 million) had only 103 practising nephrologists.^10^ Consequently, pre-end-stage renal disease care is mostly rendered by non-nephrology specialist physicians. Given the role that non-nephrology specialist physicians play in pre-end-stage renal disease care of CKD patients, it is vital to assess the quality of training that the residency programme imparts to trainee specialist care physicians with regard to CKD, especially in developing countries.

### Trends

Suboptimal CKD knowledge exists amongst non-nephrology specialists in the Western world. Published reports indicate that between 35% and 54.7% of non-nephrology specialist physicians in the USA have adequate knowledge of CKD.^11–13^ Israni and colleagues^11^ reported overall CKD knowledge of 35% amongst non-nephrology specialist physicians in the USA. Charles et al.^12^ reported that 54% of family physicians in the USA have adequate knowledge of CKD, whilst in a similar study by Agrawal et al.,^13^ only 54.7% of non-nephrology specialist physicians in the USA had adequate CKD knowledge. Seventy one per cent of physicians in the USA could correctly identify the definition of CKD.12 Boulware and colleagues,^14^ in a study that compared family physicians and non-nephrology internists, reported that 59% and 78% respectively identified the presence of CKD. Lea et al.^15^ studied non-nephrology specialist physicians with regard to identification of CKD risk factors, and found that high proportions of respondents identified diabetes and hypertension as major risk factors for CKD; however, only 34.4% identified a family history of CKD as a risk factor, compared to 76.2% in the study by Agrawal and coworkers.^13^

In Nigeria few reports exist on the knowledge and practice of physicians regarding CKD. Bosan,^16^ working in northern Nigeria, reported poor screening practices for CKD amongst primary care physicians. We recently reported that only 10% of Family Medicine residents attending a workshop in Nigeria had adequate knowledge of CKD and its screening.^17^ The majority of respondents in this study did not know the classification and staging of CKD; referrals to nephrologists were done arbitrarily and not based on any particular guidelines.

### Objectives

We embarked on this study to assess knowledge of CKD amongst examiners for the West African College of Physicians. We also sought to find out whether there were any differences in knowledge of CKD between family physicians and non-nephrology internists.

### Contribution to the field

As there is a dearth of nephrologists, non-nephrology specialist physicians render the bulk of pre-end-stage renal disease care for patients with CKD worldwide. Training and certification of non-nephrology specialist physicians in the West African sub-region is largely carried out by the West African College of Physicians, although two countries (Nigeria and Ghana) have national colleges which also carry out these functions. This study reports the adequacy of CKD knowledge amongst non-nephrology specialist physician trainers and examiners and, by extension, the quality of care that pre-end-stage renal disease patients with CKD in the West African sub-region are likely to receive.

## Ethical considerations

The study was approved by the Human Research Ethics Committee of Jos University Teaching Hospital.

### Potential benefits and hazards

The subjects were not exposed to any hazards as this was a cross-sectional study that used self-administered questionnaires. Feedback on CKD knowledge was given to the subjects.

### Recruitment procedures

Participation was voluntary and consecutive subjects who were willing to participate were recruited.

### Informed consent

Informed consent was obtained from all of the participants prior to the study.

### Data protection

Data were stored in the Microsoft Excel program, kept secure and only released for analysis when needed. Confidentiality was maintained and the anonymity of responses ensured. Personal identifiers were not collected from the subjects.

## Methods

### Materials

The questionnaire used in this study was patterned after a previously validated questionnaire used to assess knowledge of CKD.13 Domains assessed in the questionnaire included the definition, staging, risk factors, laboratory evaluation, management, complications and referral of patients with CKD. There were 30 questions in all (a blend of ‘best of five’ answers and multiple-choice questions of the ‘true or false’ style), which were used to assess knowledge of CKD. The questionnaire was also designed to obtain information on specialty, gender, practice setting, whether they had a nephrologist in their hospital, whether they saw patients with CKD, and the guidelines they used to manage patients with CKD.

### Setting

The subjects for this study were non-nephrology specialist physicians who are examiners for the West African College of Physicians. They were largely drawn from Nigeria, Ghana and Sierra Leone and constituted the bulk of the examiners. They were all holders of the Fellowship of the West African College of Physicians and actively involved in training and certification of residents in the faculties of Internal Medicine and Family Medicine. All of the examiners in the Faculty of Internal Medicine were recruited, except the nephrologists as they were excluded from the study. For every two non-nephrology specialists recruited from the Faculty of Internal Medicine, one specialist was recruited from the Faculty of Family Medicine.

### Design

This was a cross-sectional questionnaire survey of physicians who served as examiners for the faculties of Internal and Family Medicine at the Membership and Fellowship examinations of the West African College of Physicians held in Ibadan, Nigeria, on 25–30 March 2011.

### Procedure

The questionnaire was pilot tested amongst doctors in the Department of Internal Medicine at Jos University Teaching Hospital, after which modifications were made to questions and responses as appropriate. The self-administered questionnaires were distributed to the subjects and responses assessed using the National Kidney Foundation's clinical practice guidelines.^6^ Adequate knowledge of CKD was defined as answering 21 (or 70%) out of the 30 questions correctly.

### Analysis

The results are expressed as proportions for discrete variables and means ± s.d. for continuous variables. The Chi-squared test was used to compare proportions of non-nephrology internists and family physicians with regard to CKD knowledge. The Fisher's exact test was used when cells had less than five observations. The Student's *t*-test was used to compare the means of the total CKD knowledge scores of non-nephrology internists and family physicians. *P–*values ≤ 0.05 were considered significant.

## Results

### Characteristics of the study subjects

A total of 100 questionnaires were distributed, with 69 physicians (47 non-nephrology internists and 22 family physicians) returning completed questionnaires, giving a response rate of 69%. There were 56 (81.2%) men and 13 (18.8%) women. The spread of non-nephrology internists included cardiologists, infectious disease specialists, pulmonologists, endocrinologists, gastro-enterologists and neurologists. Forty-eight (69.6%) of the respondents were practising in university hospitals and 21 (30.4%) in other specialist centres. Fifty-six respondents (81.2%) had nephrologists in their hospitals, whilst 13 (18.8%) did not. Sixty three of the respondents (91.3%) attended to CKD patients in their practices, whilst 6 (8.7%) did not.

### Definition and staging of CKD

Only 19 (28.3%) of the respondents were aware of the Kidney Disease Outcome Quality Initiative guidelines for CKD management. Twenty three (34.3%) of the respondents identified the 7th Report of the Joint National Committee on Prevention, Detection, Evaluation, and Treatment of High Blood Pressure (JNC 7)^18^ as guidelines for the management of CKD. The remaining 25 respondents (37.3%) were unaware of any guidelines for the management of CKD.

Table 1 summarises the findings of our study. Only 26 (38.8%) of the respondents correctly identified CKD, defined as a positive proteinuria test twice in three months. There was no significant difference in the proportion of family physicians and non-nephrology internists who made this identification – 8 (36.4%) and 18 (38.3%) respectively; *p* = 0.87. Only 29 respondents (42%) identified stage 3 CKD as an estimated glomerular filtration rate between 30 ml/min/1.73m^2^ and 59 ml/min/1.73m^2^. Eight (36.4%) family physicians and 21 (44.7%) non-nephrology internists correctly identified this staging (*p* = 0.51).

**TABLE 1:** Performance score of family physicians (FP) and non-nephrology internists (NNIs) in West Africa regarding knowledge of CKD (*p* = 0.05).

Item of knowledge	Total		FP		NNIs		*p*-value
		
	*N*	%	*N*	%	*N*	%	
**CKD definition and classification**							
Definition of CKD	26	37.7	8	36.4	18	38.3	0.87
Classification of CKD	29	42	8	36.4	21	44.7	0.51
**Risk factors for CKD**							
Age > 60 years	41	59.4	11	50	30	63.8	0.23
Coronary artery disease	17	24.6	4	18.2	13	27.7	0.29
Diabetes mellitus	67	100	22	100	47	100	-
Daily NSAID use	63	91.3	19	86.4	44	93.6	0.28
Family history of CKD	48	69.6	15	68.2	33	70.2	0.86
Hypertension	67	100	22	100	47	100	-
Male gender	56	81.2	17	77.3	39	83	0.57
Obesity	32	46.4	13	59.1	19	40.4	0.14
SLE	62	89.9	16	72.7	46	97.9	0.003*
**Laboratory evaluation of CKD**							
eGFR	58	84.1	16	72.7	42	89.4	0.07
Urinalysis with microscopy	53	76.8	13	59.1	40	85.1	0.01*
Urine dipstick to estimate proteinuria	39	56.5	13	59.1	26	55.3	0.76
Urinary protein creatinine ratio	34	49.3	8	36.4	26	55.3	0.14
**Management of CKD**							
Target BP <130/80mmHg	35	50.7	11	50	24	51.1	0.93
ACEI/ARB for CKD	57	82.6	17	77.3	40	85.1	0.42
Cessation of smoking	54	78.3	17	77.3	37	78.7	0.89
Dietary salt restriction	54	78.3	18	81.8	36	76.6	0.62
Glycaemic control	59	85.5	19	86.4	40	85.1	0.85
Lipid control	53	76.8	15	68.2	38	80.9	0.24
Weight loss if obese	35	50.7	12	54.5	24	51.1	0.66
**Potential complications of CKD**							
Anemia	62	89.9	19	86.4	43	91.5	0.51
Bone disease	53	76.8	13	59.1	40	85.1	0.01*
Coronary artery disease	20	29	4	18.2	16	34	0.14
Dementia	18	26.1	5	22.7	13	27.7	0.66
Increased risk of diabetic complications	31	44.9	9	40.9	22	46.8	0.64
Malnutrition	27	39.1	6	27.3	21	44.7	0.16
Medication complications	35	50.7	11	50	24	51.1	0.93
Stroke	26	37.7	7	31.8	19	40.4	0.49
Referral at eGFR < 30ml/min/1.73m^2^	31	44.9	9	40.9	22	46.8	0.84
Mean CKD knowledge score (out of 30)	20 ± 5	–	18.22 ± 5.0	–	20.8 ± 5.0	–	0.05
Adequate CKD knowledge	25	36.2	6	27.3	19	40.4	0.28

ARB, angiotensin-receptor blocker; ACEI, angiotensin-converting enzyme inhibitor; BP, blood pressure; CKD, chronic kidney disease; eGFR, estimated glomerular filtration rate; NSAID, non-steroidal anti-inflammatory drugs; SLE, systemic lupus erythematosus.

* *p* = 0.05

### Risk factors for CKD

All of the respondents identified diabetes mellitus and hypertension as risk factors for CKD. Similar proportions of family physicians and non-nephrology internists identified older age, coronary artery disease, daily use of non-steroidal anti-inflammatory drugs, family history of CKD, male gender and obesity as risk factors for CKD (Table 1). There was a significant difference in the proportions of family physicians and non-nephrology internists that identified systemic lupus erythematosus as a risk factor for CKD (72.2% vs. 97.9% respectively, *p* = 0.003).

### Laboratory evaluation of CKD

Only a few of the respondents (7.2%) would check the serum creatinine level alone as a test of CKD. Fifty eight (84.1%) would check serum creatinine to estimate the glomerular filtration rate. There was no significant difference in the proportion of family physicians and non-nephrology internists in this regard (72.7% vs. 89.4%; *p* = 0.07). More non-nephrology internists identified urinalysis with microscopic examination as a test for CKD than family physicians (85.1% vs. 59.1%; *p* = 0.001). Estimation of proteinuria by the semi-quantitative (dipstick) method and protein creatinine ratio were identified by similar proportions of family physicians and non-nephrology internists (59.1% vs. 55.3%, *p* = 0.76; and 36.4% vs. 55.3%, *p* = 0.14 respectively).

### Management of CKD

A total of 61 (88.4%) respondents (81.8% of family physicians and 91.5% of non-nephrology internists; *p* = 0.24) identified the antiproteinuric effect of angiotensin-converting enzyme inhibitors/angiotensin II receptor blockers independent of blood pressure control. The target goal of blood pressure <130/80 mmHg in diabetics without proteinuria was identified by 50.7% of the respondents (50% of family physicians vs. 51.1% of non-nephrology internists; *p* = 0.93). Measures identified by the respondents as effective management for CKD included blood pressure control using angiotensin-converting enzyme inhibitors/angiotensin II receptor blockers (82.6%), glycaemic control if diabetic (85.5%), cessation of smoking (78.3%), dietary salt restriction (78.3%), control of lipid abnormalities (76.8%), and weight reduction in obese patients (50.7%).

There were no significant differences in these measures as identified by the family physicians and non-nephrology internists. Seventeen family physicians (77.3%) as against 40 non-nephrology internists (85.1%) identified the use of angiotensin-converting enzyme inhibitors/angiotensin II receptor blockers as indicated in management of CKD (*p* = 0.42). Similarly, 17 family physicians (77.3%) and 37 non-nephrology internists (78.1%) identified cessation of cigarette smoking as a useful measure in treating CKD (*p* = 0.89). Control of lipid abnormalities and weight loss (in the obese patient) were identified by 15 family physicians (68.2%) vs. 38 non-nephrology internists (80.9%; *p* = 0.24) and 12 family physicians (54.5%) vs. 24 non-nephrology internists (51.1%) respectively (*p* = 0.66).

### Potential complications of CKD

The potential complications of CKD identified by the respondents included anaemia (89.9%), bone disease (76.8%), increased risk of medication complications (50.7%), increased risk of diabetic complications (44.9%), malnutrition (39.1%), stroke (37.7%), coronary artery disease (29.0%) and dementia (26.1%). These complications were identified by similar proportions of family physicians and non-nephrology internists, except for bone disease; 19 family physicians (59.1%) and 40 non-nephrology internists (85.1%) identified bone disease as a complication of CKD; *p* = 0.01.

### Referral of CKD patients

Twenty six respondents (38.8%) were unsure of the criteria to use when referring a patient with CKD to the nephrologist; only 31 (44.9%) correctly identified the threshold of doing so based on the estimated glomerular filtration rate. The proportions of family physicians and non-nephrology internists here were similar (40.9% vs. 46.8% respectively; *p* = 0.84).

### Total CKD knowledge score

**FIGURE 1:**
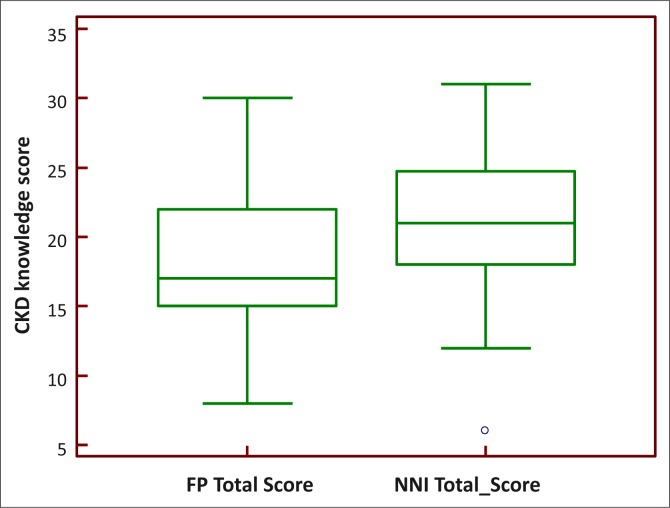
Performance score of family physicians (FP) and non-nephrology internists (NNIs) in West Africa regarding knowledge of CKD (*p* = 0.05).

The respondents got 20 ± 5 out of a total of 30 answers on CKD correct. The mean scores for the family physicians and non-nephrology internists were 18.22 ± 5.0 and 20.8 ± 5.0 respectively; *p* = 0.05, indicating a small difference reaching the border of statistical significance (Figure 1). Only 25 (36.2%) of the respondents had adequate knowledge of CKD. There was no significant difference between the proportion of family physicians and non-nephrology internists with adequate CKD knowledge, as only 6 family physicians (27.3%) and 19 non-nephrology internists (40.4%) answered 21 out of the 30 questions correctly (*p* = 0.28).

## Discussion

### Outline of the results

This study assessed the knowledge of CKD amongst examiners for the West African College of Physicians. We also sought to find out whether there was any difference in knowledge of CKD between family physicians and non-nephrology internists. We found that only a third of the non-nephrology specialist physicians who serve as trainers and examiners for a postgraduate training programme in West Africa had adequate knowledge of CKD. The level of knowledge was similar amongst family physicians and non-nephrology internists, except that the latter more frequently identified systemic lupus erythematosus as a risk factor for CKD, urinalysis with microscopy as a laboratory test for CKD evaluation, and bone disease as a complication of CKD compared to family physicians.

A third of our respondents had adequate knowledge of CKD. This parallels previous finding by Israni and colleagues,^11^ who reported overall knowledge of CKD of 35% amongst physicians in the USA. With regard to the various domains of CKD, nearly 40% of our respondents correctly defined CKD. This is comparable to the 54% reported by Charles et al.^12^ amongst family physicians and 54.7% by Agrawal et al.^13^ amongst non-nephrology specialist physicians in the USA. However, the performance of non-nephrology internists in our study with regard to definition of CKD is a far cry from that reported by Charles and coworkers^12^ amongst non-nephrology internists (38.3% vs. 71% respectively). Our findings are also much lower than the 59% and 78% performance of the family physicians and non-nephrology internists in identification of the presence of CKD and its severity as reported by Boulware and colleagues.^14^

The proportions of our respondents who demonstrated adequate knowledge in the domain of risk factors for CKD are similar to that reported by Agrawal et al.^13^ amongst internal medicine residents in the USA. The study by Lea and colleagues^15^ reported similarly high proportions of physicians identifying diabetes and hypertension as risk factors for CKD. However, only 34.4% of their respondents identified family history of CKD as a risk factor, compared to 69% in our study and 76.2% in that by Agrawal et al.^13^ Comparable performances were recorded in our study in the domains of laboratory evaluation of CKD, management of CKD and complications of CKD, with few notable differences. The proportion of physicians in our study who identified urinary protein creatinine ratio as a laboratory test to evaluate CKD was lower than in the study by Agrawal et al.^13^ (49.3% vs. 76.2% respectively). Likewise, marked differences also existed between our findings and theirs with regard to proportion of physicians identifying target blood pressure in management of non-proteinuric CKD (50% vs. 89.1%) and coronary artery disease as a complication of CKD (29% vs. 53.7%).

Proteinuria has been shown to be a risk factor for CKD progression, and its amelioration shown to retard progression of CKD.^19–22^ Over 88% of our respondents identified the antiproteinuric effect of angiotensin-converting enzyme inhibitors/angiotensin II receptor blockers. This is similar to the rates reported previously by Israni and co-workers^11^ and Agrawal et al.^13^ Use of angiotensin-converting enzyme inhibitors/angiotensin II receptor blockers forms the cornerstone of retarding progression of CKD. This is a management strategy that can be employed at all levels of care to effectively reduce progression of CKD.

### Practical implications

This study demonstrates the lack of CKD knowledge amongst non-nephrology examiners for a certifying postgraduate college in the West African sub-region. The results of our study have several implications for residency training and the quality of specialists produced by the College, as well as for clinical practice and pre-end-stage renal disease care of patients with CKD. The trainers and examiners do not have adequate knowledge of CKD. This is likely to impact on both undergraduate and postgraduate students in Medicine, the result being a similar lack of adequate CKD knowledge. This in turn is likely to affect the quality of pre-end-stage renal disease care for patients with CKD, since this is mostly offered by non-nephrology specialist physicians. Simple but effective measures like restriction of dietary salt intake, weight loss in the obese and cessation of cigarette smoking utilised in management of CKD were missed by nearly 20% – 50% of these physicians.

### Limitations of the study

We encountered some limitations that would restrict the generalisability of our findings. We studied only examiners for the West African College of Physicians, and are unaware of the knowledge of the examiners for the Ghana College of Physicians and Surgeons and National Postgraduate Medical College of Nigeria, which also contribute to postgraduate education in Ghana and Nigeria. Whilst attempting to cover all of the domains of CKD, we may have less accurately assessed the depth of knowledge, as the questionnaire utilised closed-ended questions. In addition, the definition of adequate knowledge was arbitrarily set as scoring 70% and above. Finally, the responses of the physicians cannot be readily associated with actual practice, as the knowledge-practice gap is a common limitation of questionnaire-based surveys. Despite these limitations, the strength of our report is that the physicians studied were trainers and examiners at all levels of postgraduate training in the sub-region.

### Recommendations

Educational efforts are needed to improve the CKD knowledge of non-nephrology specialist care physicians. Guidelines on CKD need to be disseminated widely amongst these physicians. Practical steps like automated reporting of estimated glomerular filtration rate should be embarked upon by laboratories in the developing world, as this may facilitate early CKD recognition and consequently appropriate referrals.

## Conclusion

This cross-sectional study assessed knowledge of CKD amongst non-nephrology specialist care physicians that serve as examiners for the West African College of Physicians. The knowledge of CKD of these physicians was inadequate, as many of them were unaware of the CKD management guidelines. There was no significant difference in the proportion of family physicians and non-nephrology internists with adequate CKD knowledge.
